# FGT-1 Is a Mammalian GLUT2-Like Facilitative Glucose Transporter in *Caenorhabditis elegans* Whose Malfunction Induces Fat Accumulation in Intestinal Cells

**DOI:** 10.1371/journal.pone.0068475

**Published:** 2013-06-24

**Authors:** Shun Kitaoka, Anthony D. Morielli, Feng-Qi Zhao

**Affiliations:** 1 Laboratory of Lactation and Metabolic Physiology, Department of Animal Science, University of Vermont, Burlington, Vermont, United States of America; 2 Department of Pharmacology, College of Medicine, University of Vermont, Burlington, Vermont, United States of America; University of Pennsylvannia, United States of America

## Abstract

*Caenorhabditis elegans* (*C. elegans*) is an attractive animal model for biological and biomedical research because it permits relatively easy genetic dissection of cellular pathways, including insulin/IGF-like signaling (IIS), that are conserved in mammalian cells. To explore *C. elegans* as a model system to study the regulation of the facilitative glucose transporter (GLUT), we have characterized the GLUT gene homologues in *C. elegans: fgt-1*, *R09B5.11, C35A11.4, F53H8.3, F48E3.2, F13B12.2, Y61A9LA.1, K08F9.1* and *Y37A1A.3*. The exogenous expression of these gene products in *Xenopus* oocytes showed transport activity to unmetabolized glucose analogue 2-deoxy-D-glucose only in FGT-1. The FGT-1-mediated transport activity was inhibited by the specific GLUT inhibitor phloretin and exhibited a Michaelis constant (*K*_*m*_) of 2.8 mM. Mannose, galactose, and fructose were able to inhibit FGT-1*-*mediated 2-deoxy-D-glucose uptake (*P* < 0.01), indicating that FGT-1 is also able to transport these hexose sugars. A GFP fusion protein of FGT-1 was observed only on the basolateral membrane of digestive tract epithelia in *C. elegans*, but not in other tissues. FGT-1::eGFP expression was observed from early embryonic stages. The knockdown or mutation of *fgt-1* resulted in increased fat staining in both wild-type and *daf-2* (mammalian insulin receptor homologue) mutant animals. Other common phenotypes of IIS mutant animals, including dauer formation and brood size reduction, were not affected by *fgt-1* knockdown in wild-type or *daf*-2 mutants. Our results indicated that in *C. elegans*, FGT-1 is mainly a mammalian GLUT2-like intestinal glucose transporter and is involved in lipid metabolism.

## Introduction

Glucose is an essential energy source and substrate for the synthesis of macromolecules in most, if not all, living organisms. In mammalian cells, glucose is taken up by two families of glucose transporters that are located in the cell plasma membrane. The facilitated glucose transporters (GLUT, gene symbol: SLC2A) mediate passive glucose diffusion across the plasma membrane in most tissues, whereas Na^+^/glucose cotransporters (SGLT, gene symbol: SLC5A) mediate Na^+^-dependent secondary active glucose transport mainly in the epithelial cells of the small intestine and kidney convoluted tubules [1,2].

The GLUT family contains 14 members that have high sequence similarities and share common structure characteristics, including 12 transmembrane domains and sugar transporter signatures [2,3]. However, individual transporters have different transport kinetics, tissue distribution and regulatory properties [2]. Among the 14 members, class I GLUTs (GLUT1-4) are well-studied because of their physiological and pathophysiological roles in cells. GLUT1 is ubiquitously expressed and mediates basic glucose uptake. GLUT2 is mainly localized in the plasma membrane of hepatic cells and in the basolateral membrane of the luminal epithelial cells of the intestine and kidney convoluted tubules [4]. In intestinal and kidney cells, GLUT2 is responsible for releasing the glucose that is absorbed or reabsorbed by SGLT1 or SGLT2 in the apical membrane into the bloodstream [2,5,6]. GLUT3 is known as a neuron-specific glucose transporter and is mainly expressed in the brain [7,8]. GLUT4 mediates insulin-regulated glucose uptake in muscle cells and adipocytes. In these cells, insulin stimulates the translocation of GLUT4 from the intracellular pool to the plasma membrane and thus, increases glucose uptake and utilization [9,10]. This regulation plays a critical role in maintaining whole-body glucose homeostasis. GLUTs belong to the major facilitator superfamily (MFS), which consists of members that are present ubiquitously in bacteria, archaea, cyanobacteria, fungi, protozoa, plants and animals.

*C. elegans* is an attractive animal model for biological and biomedical research because of its small size, simplicity, short lifespan (21 days) and quick turn over (3 days), ease of propagation and maintenance, routine genetic manipulations, and cost-effectiveness. It has been widely used as a model system for studying aging, reproduction, metabolism, and other physiological processes that are known to be regulated by insulin/IGF-like signaling (IIS). IIS is well-conserved in *C. elegans*, and many mammalian insulin signaling molecule homologues have been identified (e.g., DAF-2 is the *C. elegans* counterpart of the mammalian insulin receptor) and studied in *C. elegans*. However, there have been no GLUT homologues functionally identified in *C. elegans*, and no studies have been published yet about IIS regulation of glucose uptake in *C. elegans*.

To explore *C. elegans* as a model system for studying the regulation of glucose transporter functions, especially by IIS, we have characterized the potential *C. elegans* GLUT homologues, *fgt-1*, *R09B5.11, C35A11.4, F53H8.3, F48E3.2, F13B12.2, Y61A9LA.1, K08F9.1 and Y37A1A.3*, that show high sequence homologies to human GLUTs.

## Results

### 1. Bioinformatic analysis of GLUT homologues in the *C. elegans* genome

**Figure 1:**
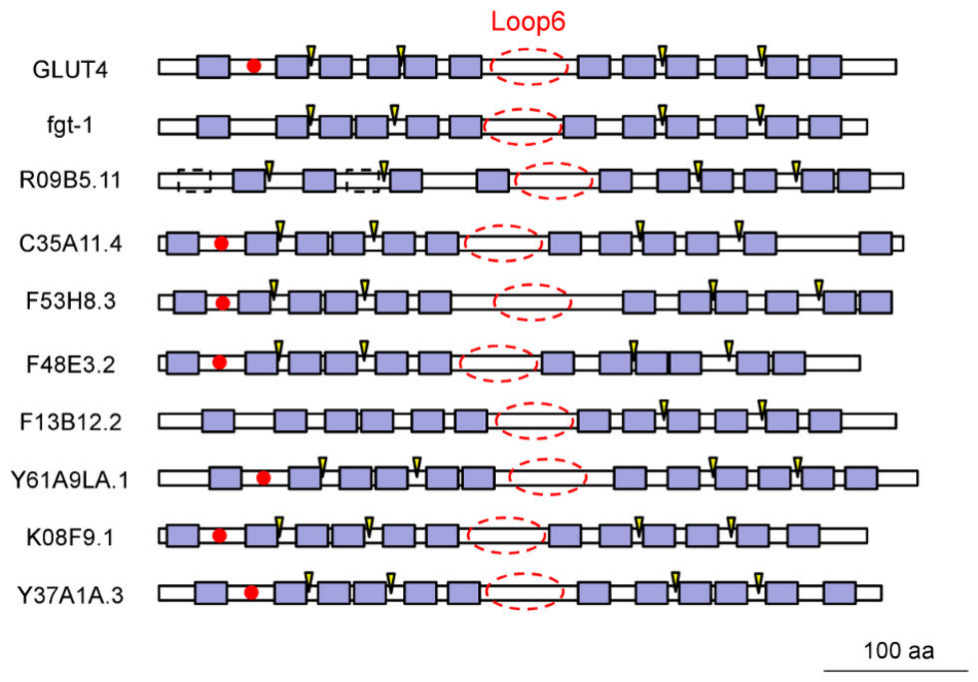
Structural schematic representation of GLUT candidate genes in *C. elegans*, compared with human GLUT4. The amino acid sequence of individual *C. elegans* genes was obtained from Wormbase (http://www.wormbase.org/). The blue boxes indicate the predicted transmembrane domains by Wormbase, and the dashed boxes in *R09B5.11* indicate the missing predicted transmembrane domains. Red filled circles indicate potential N-glycosylation sites that were predicted by NetNGlyc (http://www.cbs.dtu.dk/services/NetNGlyc/). Arrowheads indicate known functionally important residues that were found in human GLUT4: R92, R153, R333/4, and E393 [11]. The predicted conserved long loop 6 is indicated by red dashed circles.

BLASTP searches were performed using protein sequences of human GLUT1 through 12 genes against the *C. elegans* database (Wormbase: http://www.wormbase.org/). Forty seven genes were found to have higher scores than a cut off E-value of 1E-2 (Table S1). After removing those candidates with known functions other than sugar transport, the remaining candidates were analyzed for facilitative glucose transporter signatures, including 12 transmembrane domains [3], an N-glycosylation site either within the first or sixth extracellular loop and several highly conserved residues [2]. Nine genes, including *fgt-1*, *R09B5.11*, *C35A11.4*, *F53H8.3*, *F48E3.2*, *F13B12.2*, *Y61A9LA.1*, *K08F9.1*, and *Y37A1A.3*, passed these analyses and were considered initial *C. elegans* GLUT (ceGLUT) candidates (Figure 1). FGT-1 and R09B5.11 had the highest homologies to the class I family of human GLUTs (hGLUTs) compared with the other candidates. The BLASTP E-values of FGT-1 and R09B5.11 against hGLUT1 or hGLUT4 were 7E-82, 1E-66, 3E-84 and 6E-67, respectively, which were much lower than other candidates (> 2E-36 and > 5E-31 to hGLUT1 and hGLUT4, respectively) (Table S1). R09B5.11 was predicted to lack two potential transmembrane domains (Figure 1) but remained in the candidate list because of its high E-values in our BLASTP analysis.

**Figure 2:**
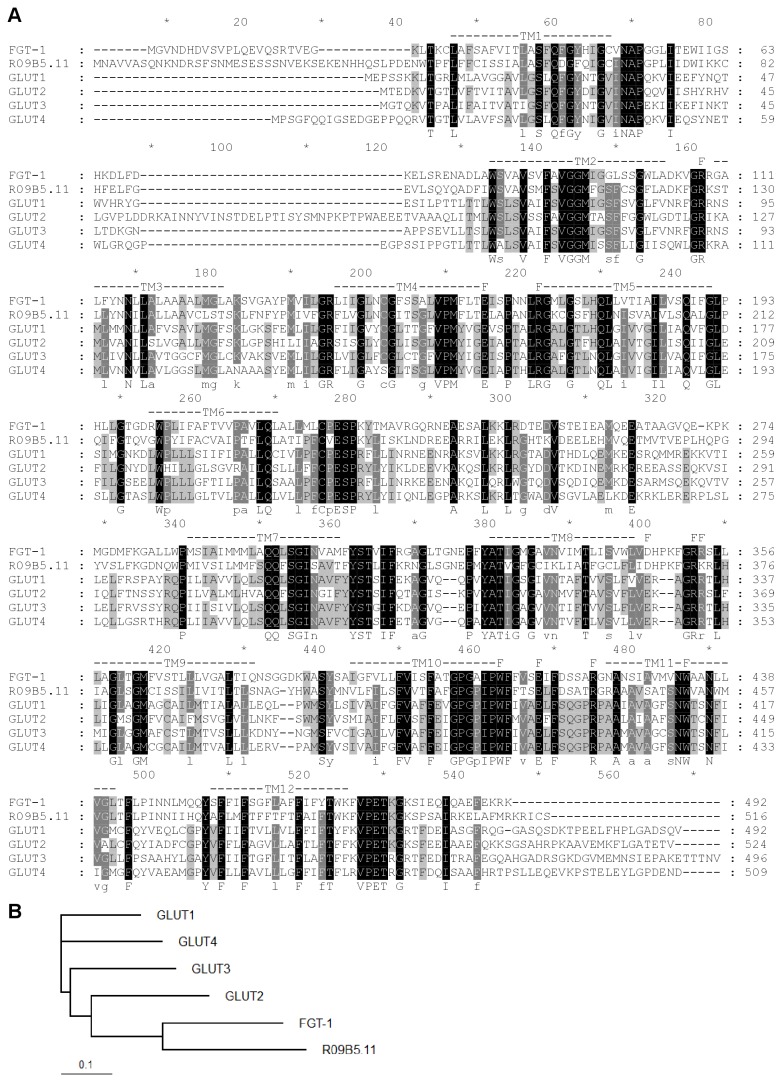
Amino acid sequence alignments of human GLUT1-4 and *C. elegans* FGT-1 and R09B5.11. A. Alignments of the deduced amino acid sequences of FGT-1, R09B5.11 and human GLUT1-4 were performed with the Clustal W program with open gap penalty = 10 and gap extension penalty = 0.05. Residues that are highlighted by a black shaded background represent absolutely conserved amino acids, and the gray shaded background indicates four or more conserved residues at those positions. Regions of presumed transmembrane domains (TM) [32] are indicated by numbered dashed lines, and the functionally important residues for glucose uptake activity are indicated by the letter “F” at the top of the sequence alignments. In addition, the highly conserved amino acids are shown on the bottom of the sequence alignment. B. Phylogenetic tree of the aligned sequences in (A) by the Clustal W program. Scale bar indicates relative branch lengths obtained from the Clustal W alignment result.

The deduced amino acid sequences of all 9 ceGLUT candidates were aligned with the class I family of hGLUTs in Figure S1. Figure 2A shows the higher resolution alignments of FGT-1 and R09B5.11 with hGLUT1-4. These alignments showed that many residues were well-conserved in these sequences, including those in the transmembrane domains (TM) 1 and 5, which were predicted to be absent in R09B5.11, and some residues that are known to be functionally important in hGLUTs, such as R92, E146, R153, E329, R333/4, W388, E393, R400, and W412 as reported by Schurmann et al. (2007) [11]. Moreover, they all contained deduced sugar transporter domains and major facilitator domains (PFAM: http://pfam.sanger.ac.uk/). A phylogenetic tree drawn by the alignment showed that hGLUT2 was the closest isoform for all ceGLUT candidates, and that FGT-1 and R09B5.11 were the closest homologues to human class I GLUTs (Figure 2B and Figure S2).

Thus, we pursued the functional characterization of all of these 9 ceGLUT candidates. We first cloned the full-length cDNAs of these *C. elegans* genes by PCR using specific primers that are in the 5’- or 3’-untranslated regions of corresponding genes based on the sequence information in Wormbase. In the database, *fgt-1* was predicted to have two mRNA variants, *fgt-1a* and *fgt-1b*, which have differences in the first exon, that result in different N-termini of the protein. We cloned *fgt-1a* cDNA for this study because *fgt-1a* showed a slightly lower E-value than *fgt-1b* in our BLASTP search against the class I family of hGLUTs (Table S1).

### 2. Glucose transport activity of ceGLUT candidates

**Figure 3:**
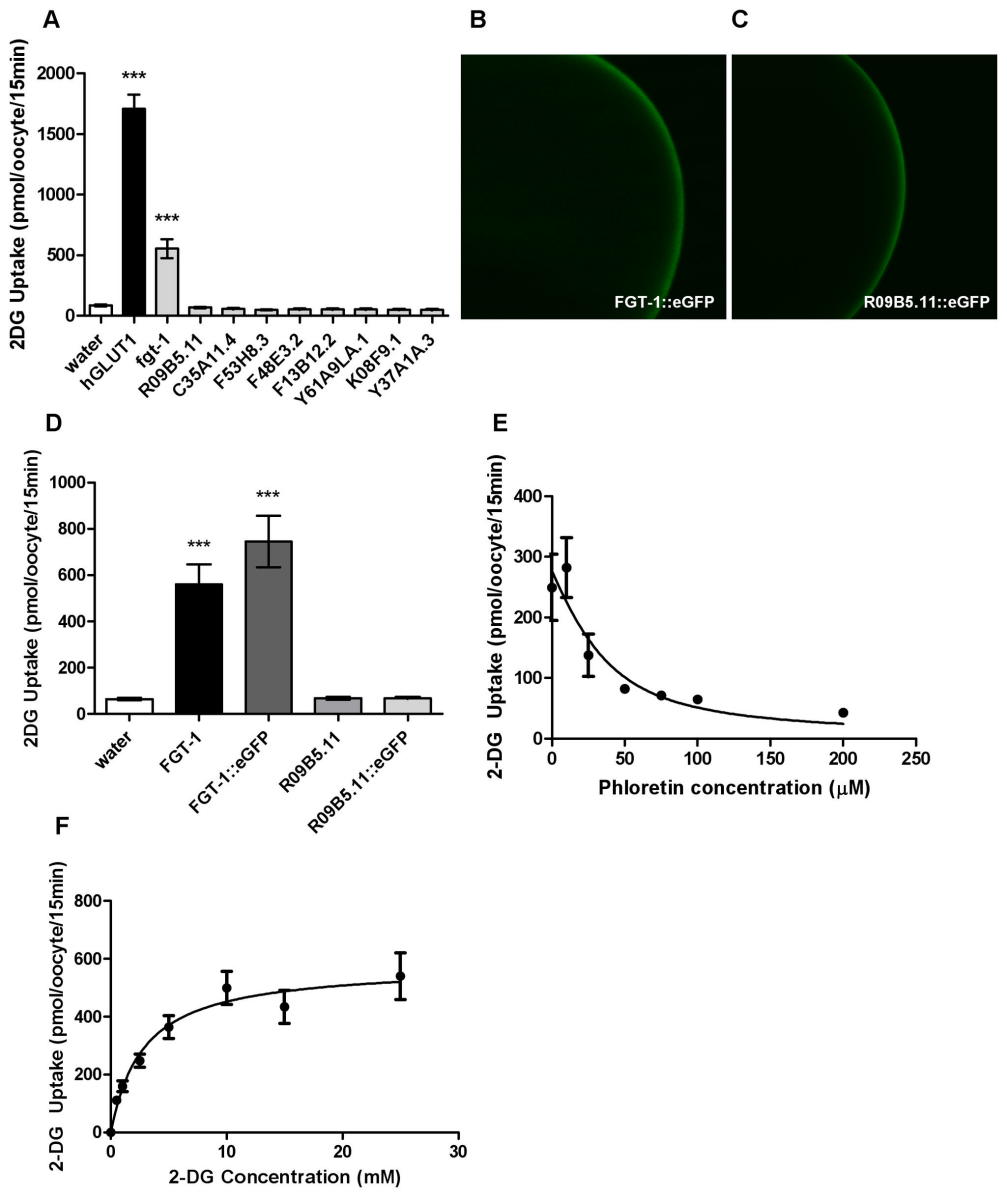
Glucose transport activity and kinetics of *C. elegans* GLUT candidates in *Xenopus* oocytes. A. Analysis of 2-deoxy-D-glucose (2-DG) transport activities of FGT-1, R09B5.11, C35A11.4, F53H8.3, F48E3.2, F13B12.2, Y61A9LA.1, K08F9.1, and Y37A1A.3 in *Xenopus* oocytes. Water or the cRNA of *C. elegans* genes or hGLUT1 was injected into oocytes, and the oocytes were incubated in 10 mM 2-DG containing 2-deoxy-D-[1-^3^H]-glucose ( ^3^H-2-DG) for 15 min before being counted. Error bars represent SEM (n ≥ 31). Statistical analysis was conducted using Dunnett’s one-way ANOVA before the Tukey-Kramer HSD test, with water-injected samples as the control group (***P < 0.001). B and C. Plasma membrane localization of FGT-1 and R09B5.11. The eGFP fusion proteins of FGT-1 (B) or R09B5.11 (C) were individually expressed in oocytes and were observed under a confocal laser microscope. D. Analysis of 2-DG transport activities of the FGT-1::eGFP and R09B5.11::eGFP fusion proteins in *Xenopus* oocytes. Water or the cRNA of *fgt-1, fgt-1::egfp, R09B5.11* or *R09B5.11::egfp* was injected into oocytes, and the oocytes were incubated in 10 mM 2-DG containing ^3^H-2-DG for 15 min before being counted. Error bars represent SEM (n ≥ 27). Statistical analysis was conducted using Dunnett’s one-way ANOVA before the Tukey-Kramer HSD test, with water-injected samples as the control group (***P < 0.001). E. Inhibition of the glucose transport activity of FGT-1 by phloretin. The *fgt-1 cRNA-injected* oocytes were incubated in 10 mM 2-DG with ^3^H-2-DG and increasing concentrations of phloretin from 0 µM to 200 µM for 15 min. The 2-DG uptake of *fgt-1* cRNA-injected oocytes was corrected by the uptake of water-injected oocytes. Error bars represent SEM (n = 10). F. Kinetic analysis of 2-DG uptake by FGT-1. The *fgt-1* cRNA- or water-injected oocytes were exposed to increasing concentrations of 2-DG (0-30 mM) containing ^3^H-2-DG for 15 min. Points represent 2-DG uptake of *fgt-1* cRNA-injected oocytes after correction for the uptake of water-injected oocytes. Error bars represent SEM (n ≥ 13). Michaelis-Menten nonlinear analysis was conducted in GraphPad Prism 5 (GraphPad Software Inc., La Jolla, CA).

The functional glucose transport activities of ceGLUT candidates were assessed in *Xenopus laevis* oocytes. Synthesized cRNA of individual ceGLUT candidates was injected into oocytes, and the oocytes were then incubated in 10 mM 2-deoxy-D-glucose (2-DG) containing 1 µCi 2-deoxy-D-[1-^3^H]-glucose ( ^3^H-2-DG) for 15 min. The hGLUT1 cRNA-injected oocytes were used as a positive control, and the water-injected oocytes were used to determine the endogenous levels of glucose uptake in the oocytes. FGT-1 showed significant 2-DG transport activity, although the activity appeared much lower than hGLUT1, whereas all other gene products that were examined showed no activity for 2-DG transport (Figure 3A). We confirmed the plasma membrane localization of FGT-1 and R09B5.11 in the oocytes by the injection of GFP fusion constructs of FGT-1 and R09B5.11 into oocytes. Both FGT-1::eGFP and R09B5.11::eGFP were localized in the plasma membrane of oocytes (Figure 3B, C). The eGFP fusion protein of FGT-1 showed the same 2-DG uptake activity as non-fusion FGT-1, whereas the R09B5.11::eGFP fusion protein failed to show activity, similar to our results for untagged R09B5.11 (Figure 3D). These results indicated that although the R09B5.11 is localized to the plasma membrane, it has no transport activity of glucose, unlike FGT-1.

Because facilitative glucose transport is known to be inhibited by the specific inhibitor phloretin, we examined whether the glucose transport activity of FGT-1 is inhibited by phloretin. The *fgt-1* cRNA-injected oocytes were incubated in 2-DG solution with increasing concentrations of phloretin from 0 µM to 200 µM. Phloretin significantly inhibited FGT-1-mediated 2-DG uptake with concentrations of 50 µM or higher (*P* < 0.01, n = 10) (Figure 3E). Non-linear one phase decay analysis estimated an inhibitory plateau at 277 pmols/oocyte/15 min.

We further performed the transport kinetic analysis of FGT-1 to 2-DG with increasing concentrations of 2-DG (Figure 3F). The *K*_*m*_ value of FGT-1 for 2-DG was determined to be 2.8 mM.

### 3. Hexose substrate specificity of FGT-1

**Figure 4:**
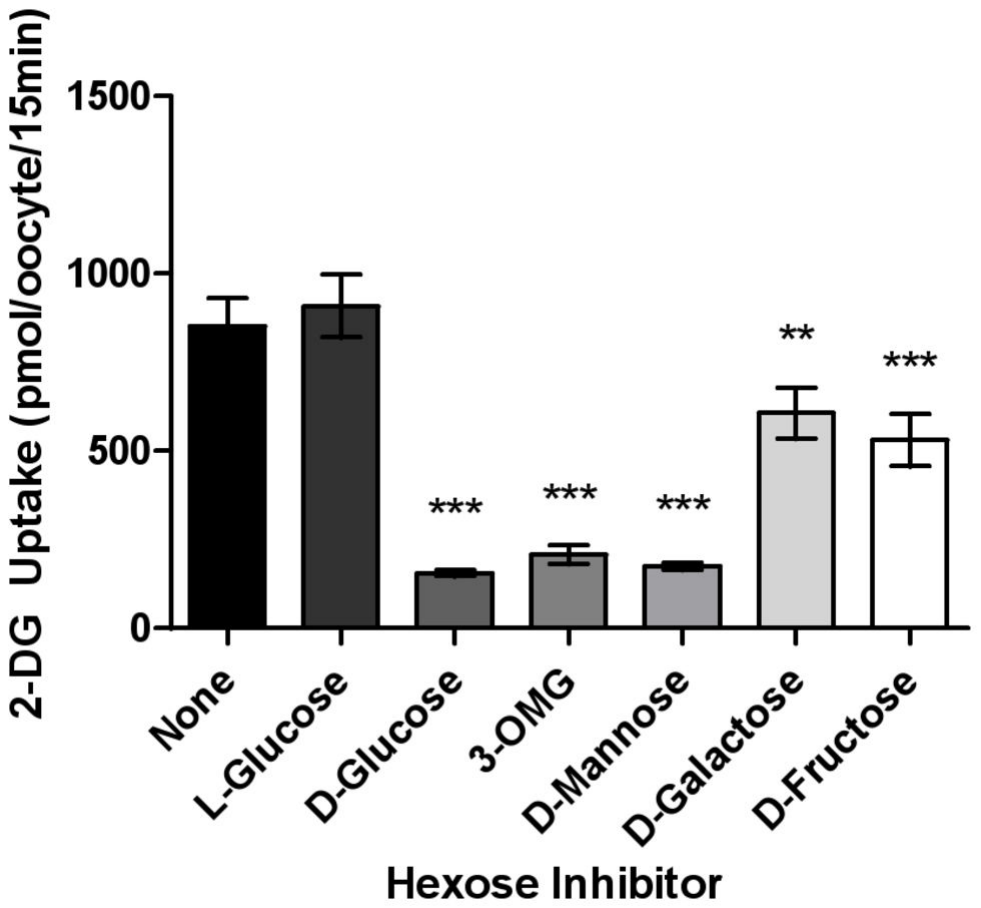
Hexose sugar substrate specificity of fgt-1. Oocytes injected with either fgt-1 cRNA or water were exposed to 10 mM 2-DG containing ^3^H-2-DG and 30 mM concentrations of L-glucose, D-glucose, 3-O-methylglucose (3-OMG), D-mannose, D-galactose, or D-fructose for 15 min. 2-DG uptake from water-injected oocytes was subtracted from *fgt-1* cRNA-injected oocytes. Error bars represent SEM (n = 40). Statistical analysis was conducted using Dunnett’s one-way ANOVA before the Tukey-Kramer HSD test, with L-glucose as the control group (**P < 0.01, ***P < 0.001).

The substrate specificity of FGT-1 to different hexose sugars was analyzed by the competitive inhibition of mannose, galactose, and fructose on 2-DG uptake of FGT-1 in oocytes (Figure 4). The inhibition effects of hexose sugars were compared with that of L-glucose, which is not transported by GLUTs [12]. Indeed, L-glucose showed no inhibition of the 2-DG uptake by FGT-1 compared with the uptake without addition of any hexose sugar (Figure 4). The 2-DG uptake by FGT-1 was significantly inhibited by D-glucose (79% inhibition), 3-*O*-methylglucose (77%), D-mannose (79%), D-galactose (33%), or D-fructose (42%) (*P* < 0.01, n = 40, Figure 4). These results indicated that FGT-1 is able to transport D-mannose as well as D-glucose and is also able to transport D-fructose and D-galactose with lower activities.

### 4. Cellular and subcellular localizations of FGT-1 and R09B5.11 in *C. elegans*

**Figure 5:**
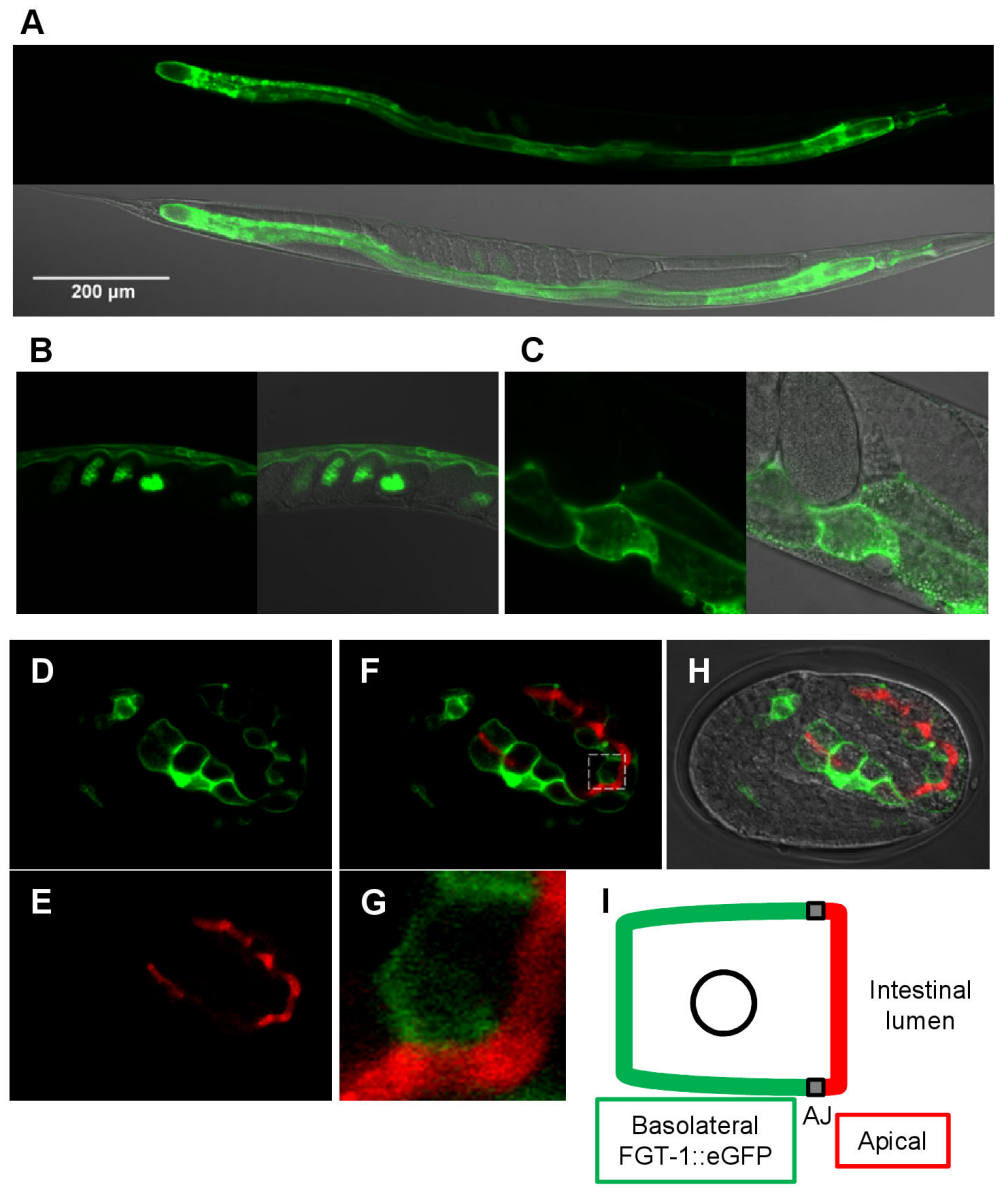
Cellular and subcellular localizations of FGT-1 in *C. elegans*. The *fgt-1::egfp* fusion construct under the control of the *fgt-1* promoter was injected into *C. elegans*, and the expression of the FGT-1::eGFP fusion protein was visualized with a confocal laser microscope. A: whole animal, B: embryos inside of a parental worm, C: higher magnification image of mid-body. For each panel (A to C), GFP fluorescence was shown alone or as a merged picture with differential interference contrast (DIC) images. D–H. Immunostaining of the apical membrane marker IFB-2 for *fgt-1::egfp* embryos. Subcellular localizations of FGT-1::eGFP (green) and IFB-2 (red) were observed under a confocal laser microscope. D: FGT-1::eGFP, E: IFB-1 immunostaining. F: Merged image of D and E.G: The higher magnification of the white box area in F.H: Merged image of eGFP fluorescence and DIC images. I. Schematic diagram of an intestinal cell with subcellular localizations of eGFP (green) and IFG-2 (red). The potential adherent junction (AJ) is indicated by a dark box.

To study the specific physiological functions of FGT-1, we built the *fgt-1::egfp* fusion construct under the control of 2 kb upstream promoter sequences of *fgt-1*. The plasmid was injected into *C. elegans*. The expression of FGT-1::eGFP fusion protein was observed in pharyngeal muscle and intestinal cells (Figure 5A). The expression was observed initially in early embryonic stages (Figure 5B). In adult animals, FGT-1::eGFP was mainly localized in the plasma membrane of intestinal cells (Figure 5C). To determine the apico-basal polarity of FGT-1::eGFP subcellular localization, we immunostained the *fgt-1::egfp* animal embryos with an antibody to the apical membrane marker IFB-2, which is an intermediate filament protein and a component of the terminal web on the apical domain of intestinal cells [13,14]. FGT-1::eGFP was not co-localized with IFB-2, suggesting that FGT-1::eGFP was only expressed on the basolateral membrane, but not on the apical membrane (Figure 5D–I). In addition, FGT-1::eGFP localization was not affected by fasting (data not shown).

**Figure 6:**
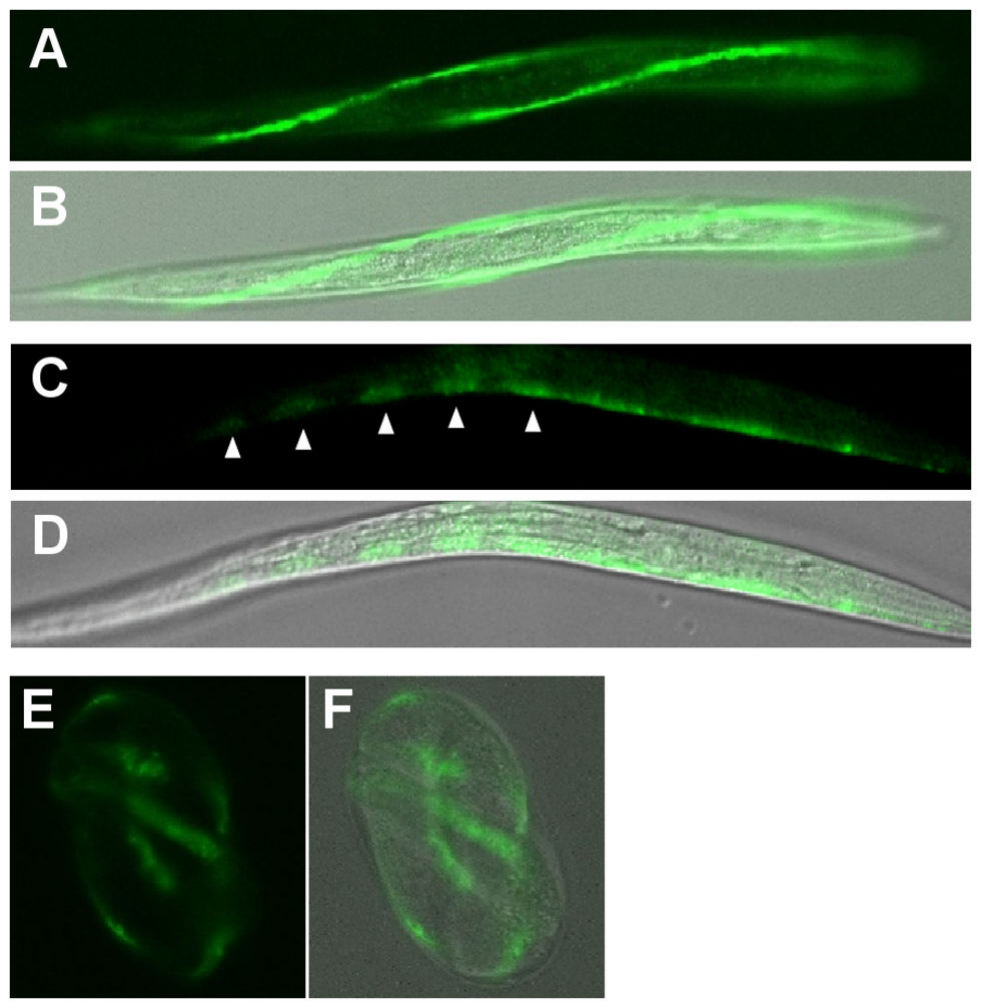
Cellular localizations of R09B5.11 in *C. elegans*. The *R09B5.11::egfp* fusion construct under the control of the *R09B5.11* promoter was injected into *C. elegans*, and the expression of the R09B5.11::eGFP fusion protein was visualized with a confocal laser microscope. GFP images and merged pictures of GFP with DIC images in the L2 stage (A–D) or three-fold embryo (E and F) are shown. Left- and right-lateral strings of seam cells (A and B) and individual seam cells (C and D) are visualized. Arrowheads indicate each seam cell.

Expression of R09B5.11::eGFP was also observed in *C. elegans*. R09B5.11::eGFP was expressed from the early embryonic stage through the L2 stage and appeared to be expressed in seam cells (Figure 6).

### 5. In vivo functional analysis of FGT-1 in *C. elegans*

**Figure 7:**
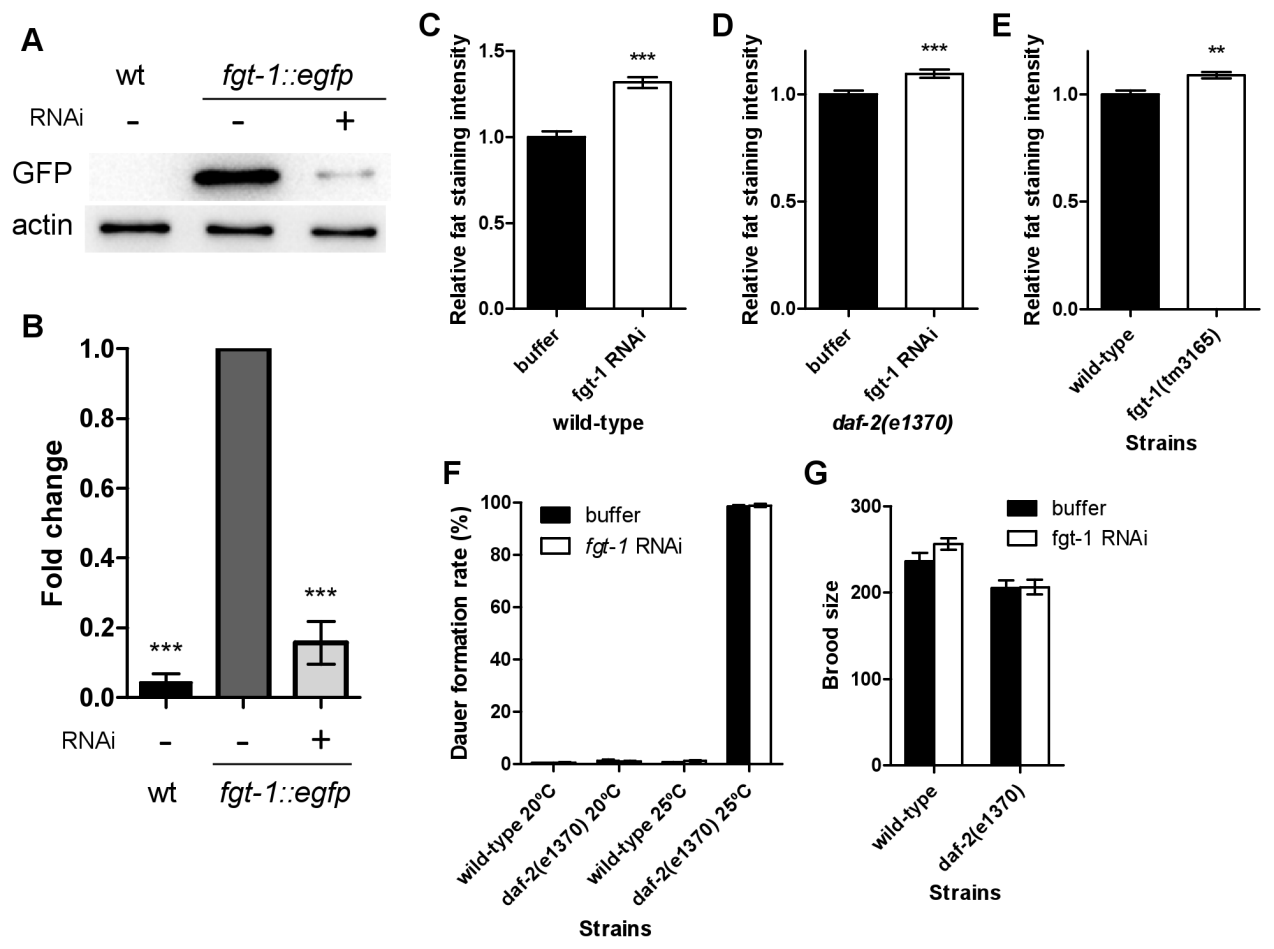
Functional analyses of fgt-1 on fat storage, dauer formation and brood size in *C. elegans*. A. The expression of the *fgt-1::egfp* fusion protein was detected by Western blot analysis using an anti-GFP antibody in the wild-type worms (wt) injected with (+) or without (-) the *fgt-1* double-stranded RNA (dsRNA, RNAi). B. The intensities of the fusion protein in A were quantified by Image Lab Software and were normalized to β-actin levels. Error bars represent SEM (n = 4). C, D, and E. Fat accumulation in the *fgt-1* RNAi wt (C) and *daf-2*(*e1370*) mutant worms (D), as well as in *fgt-1*(*tm3165*) mutant animals (E, no RNAi), was measured by Sudan black B staining. Staining intensity was quantitated using ImageJ software (NIH). Error bars represent SEM (n ≥ 41). Statistical analysis was conducted using Student’s *t*-test (**P < 0.01, ***P < 0.001). F and G. Comparison of the effects of *fgt-1* knockdown on the dauer formation rate (F) and brood size (G). In F, wild-type and *daf-2*(*e1370*) worms were incubated at either 20 ^°^C or 25 ^°^C. Error bars represent SEM (n ≥ 9 and 25 for F and G, respectively). Statistical analysis was conducted using Student’s *t*-test.

Because FGT-1 is mainly located in intestinal cells, which are the major site of fat accumulation in *C. elegans*, we studied the role of *fgt-1* in fat accumulation. We injected into *C. elegans* 112 bp double-stranded *fgt-1* RNA (dsRNA) that was synthesized from exon 6 of the *fgt-1* gene, which has limited sequence homologies to other GLUT homologues. The *fgt-1* mRNA levels that were measured by real-time RT-PCR did not show a significant reduction of *fgt-1* mRNA in the *C. elegans* that were injected with *fgt-1* dsRNA (data not shown). Due to the lack of the antibodies to FGT-1, we confirmed the RNAi effect on *fgt-1* protein levels by injecting *fgt-1* dsRNA into *fgt-1::egfp* transgenic animals. F1 progeny were analyzed by Western blot analysis using an anti-GFP antibody. In dsRNA-injected transgenic animals, the FGT-1::eGFP protein level was decreased to 16% of that in the animals injected with buffer, confirming the knockdown effect of our RNAi (Figure 7A, B).

The *fgt-1* dsRNA was then injected into wild-type and *daf-2* mutant animals, and fat was then stained in these animals with Sudan black B. As shown in Figure 7C and 7D, fat staining intensity in the wild-type and *daf-2* mutant *C. elegans* injected with *fgt-1* RNAi was 139% and 109%, respectively, compared with the control animals (*P* < 0.001, n > 47). We further confirmed this result in an *fgt-1*(*tm3165*) mutant animal that showed 9% higher fat storage compared with wild-type animals (*P* < 0.01, N = 41) (Figure 7E).

In addition, we analyzed the effects of *fgt-1* RNAi on dauer formation and brood size. For both phenotypes, *fgt-1* RNAi had no significant effect on wild-type and *daf-2*(*e1370*) animals (Figure 7F, G). Increasing the cultivation temperature from 20 °C to 25 °C, which led to strong dauer formation in *daf-2*(*e1370*), also had no effects on dauer formation by *fgt-1* RNAi in both wild-type or *daf-2*(*e1370*) animals (Figure 7F).

## Discussion

BLASTP searches of the *C. elegans* genome with hGLUT cDNAs and structural analyses resulted in nine *C. elegans* GLUT candidate genes: *fgt-1*, *R09B5.11*, *C35A11.4*, *F53H8.3*, *F48E3.2*, *F13B12.2*, *Y61A9LA.1*, *K08F9.1*, and *Y37A1A.3*. Among them, *fgt-1* and *R09B5.11* displayed the highest sequence homologies to the class I family of hGLUTs. Thus, in this study, we aimed to characterize the glucose transport properties of these proteins and to study their physiological functions in *C. elegans*. The glucose transport properties of all 9 ceGLUT candidates were first assayed in a *Xenopus* oocyte model, which is broadly used for the measurement of glucose transporter activity because of its low endogenous glucose transport level [15]. Although FGT-1 showed significant glucose transport activity, no such activity was observed for any of the other candidates. Because R09B5.11 was localized on the plasma membrane in oocytes, our results indicated that unlike FGT-1, R09B5.11 is not a facilitative glucose transporter in *C. elegans*. The inability of R09B5.11 to transport glucose may be explained by its structural analysis. Although R09B5.11 has high sequence homologies to GLUTs and FGT-1 (55% to hGLUT1 and 62% to FGT-1), it is predicted to have only 10 TMs, rather than the 12 TMs predicted in all hGLUTs [2,3] and FGT-1. R09B5.11 may lack one or two TMs that are required for glucose transport function or all 12 TMs may be essential [3]. However, R09B5.11 showed expression in the early developmental stage and may play a role in development. Although R09B5.11 had no transport activity for glucose, it may have transport activity for other substrates. Similar to R09B5.11, all of the other 7 candidates showed no transport activity for glucose, however, we cannot completely exclude the possibility that these proteins transport glucose because we have not examined their subcellular localizations. If a protein is localized intracellularly in our experimental conditions, its transport activity would not be revealed in our analysis.

Because FGT-1 showed glucose transport activity, we further analyzed its transport properties and kinetics. The transport activity of FGT-1 can be inhibited by a facilitative glucose transport specific inhibitor phloretin [16] in a dose-dependent manner. Kinetic assays revealed that FGT-1 has a *K*_*m*_ of 2.8 mM for 2-DG, a non-metabolized glucose analog. This *K*_*m*_ value is much lower than the *K*_*m*_ reported for hGLUT1 (11.7 mM) or hGLUT4 (4.6 mM) [17,18], which is not surprising because *C. elegans* lives in a low glucose soil environment and may require cells to have transporters with a high affinity for glucose for its acquisition. In addition, we showed that the 2-DG transport activity of FGT-1 can be inhibited by not only D-glucose and the glucose analog 3-OMG but also by several other hexose sugars, including D-mannose, D-galactose, and D-fructose. It appears that FGT-1 can transport D-mannose equally as well as D-glucose but has relatively low activities for D-fructose and D-galactose.

To study the *in vivo* physiological functions of FGT-1 in *C. elegans*, we first investigated its cellular and subcellular localizations in whole animals using the FGT-1::eGFP fusion protein controlled by the *fgt-1* promoter. Because the eGFP fusion protein of FGT-1 showed the same 2-DG uptake activity as non-fusion FGT-1 in our oocyte assay, FGT-1::eGFP should reflect the native localization of FGT-1 in *C. elegans*, although experiments to determine if FGT-1::eGFP can functionally rescue *fgt-1* mutant phenotypes are needed to fully support this conclusion. FGT-1 is observed to be mainly distributed to the basolateral membrane of intestinal cells. In mammals, intestinal glucose absorption is mainly mediated by the Na^+^/glucose cotransporter SGLT1 located on the apical membrane [19]. It is not known whether *C. elegans* uses the same mechanism, and an SGLT1 homologue has not been reported in *C. elegans*. A BLASTP search using human SGLT sequences showed no SGLT homologous genes in *C. elegans*. Nevertheless, it is likely that glucose is also absorbed from the intestinal lumen by an active glucose transporter in *C. elegans*. FGT-1 may play a role similar to mammalian GLUT2, which is responsible for releasing absorbed glucose from the intestinal cells to interstitial fluid [2,5,6]. Supporting this idea, our phylogenetic tree analysis indicates that hGLUT2 is the closest human class I GLUT isoform to FGT-1.

We next investigated the effects of the knockdown of *fgt-1* on multiple physiological phenotypes in *C. elegans*. We found that fat staining was increased in *fgt-1* knockdown and mutant worms. This observation is consistent with our observation that FGT-1 is an intestinal transporter because the intestine is the major site of lipid storage in *C. elegans* [20,21]. This result also suggests a role for FGT-1 in lipid metabolism in *C. elegans*. However, it is surprising that the knockdown or mutation of *fgt-1* resulted in increased fat staining because this result indicates that the knockdown or mutation of *fgt-1* results in either an increased lipogenesis or a decreased lipolysis or a combination of both. Because glucose is a substrate and an energy source for lipid synthesis, increased fat staining in *fgt-1* knockdown and mutant animals implies that glucose is not limited in these cells. This observation is consistent with our hypothesis that FGT-1 may be involved in the release of absorbed glucose from intestinal cells and not in glucose absorption. Malfunctions of FGT-1 may reduce glucose release and increase intracellular glucose levels in intestinal cells, leading to increased lipid synthesis.

We did not observe any effects of knockdown of *fgt-1* on dauer formation and brood size, indicating that FGT-1 does not play an essential role in the overall energy metabolism and reproductive function of *C. elegans*. *C. elegans* undergoes dauer larva formation when nutrients become limited [22]. Thus, the knockdown of *fgt-1* appears to not have a detrimental effect on energy metabolism in *C. elegans*, which is also consistent with our observation that FGT-1 localizes to intestine cells and may not be responsible for glucose uptake in other tissues and cells in *C. elegans*. Other glucose transporter isoforms may have to be expressed in other tissues and cells to mediate glucose uptake. Another explanation is that *C. elegans* cells may be able to efficiently use other energy sources, such as glycerol, to replace glucose [23].

It is well studied that insulin stimulates GLUT4 translocation in mammalian adipose cells and muscle cells [9,10]. During fasting, GLUT4 is mainly located intracellularly, whereas after feeding, the rising insulin levels in the blood stimulate GLUT4 translocation to the plasma membrane which increases glucose uptake by the cells. We investigated the possibility that IIS may regulate FGT-1 subcellular localization in *C. elegans* by examining the subcellular localization of FGT-1::eGFP in the fasting condition. The localization of the fusion protein showed no changes compared with the non-fasting condition. In addition, because dauer formation and reproduction, as well as fat staining, are known to be regulated by IIS in *C. elegans*, and because none of them were affected by the knockdown of FGT-1 in both wt and the *daf-2* mutant in this study, our data suggest that FGT-1 may not be involved in IIS regulation of these processes. However, our data in *daf-2*(*e1370*) were confounded by the non-null nature of the allele. Further studies with different IIS signaling mutants, such as *daf-16* (a mammalian FOXO homologue), and with different culture conditions (such as temperature and calorie restriction) are required to make this conclusion.

In summary, in this study we have identified FGT-1 as a mammalian GLUT counterpart in *C. elegans*. FGT-1 is a digestive tract-specific isoform that is located in the basolateral membrane of intestinal epithelial cells. Thus, FGT-1 may function like mammalian GLUT2 and serve to release glucose from intestinal cells to the interstitial fluid. The knockdown or mutation of *fgt-1* increases fat storage in intestinal cells, indicating a role of FGT-1 in lipid metabolism in *C. elegans*.

## Materials and Methods

### *C. elegans* strains and culture

The *C. elegans* strains used in this study were obtained from the Caenorhabditis Genetics Center (http://www.cbs.umn.edu/cgc). The strains used were wild-type N2 and *daf-2*(*e1370*). The *C. elegans* strains were cultivated at 20 °C under standard conditions [24].

### Plasmid constructions

PCR primers that were used for all plasmid constructions are listed in Table 1. PCR reactions in plasmid constructions were all carried out using the high fidelity Platinum *Pfx* DNA polymerase (Invitrogen) unless otherwise specified. The full-length cDNA of individual genes was amplified from the cDNA library of wild-type worms with the following primer sets: fgt1-5utr and fgt1-3utr for *fgt-1*, R09-5utr and R09-3utr for *R09B5.11*, C35-5utr and C35-3utr for *C35A11.4*, F53-5utr and F53-3utr for *F53H8.3*, F48-5utr and f48-3utr for *F48E3.2*, F13-5utr and F13-3utr for *F13B12.2*, Y61-5utr and Y61-3utr for *Y61A9LA.1*, K08-5utr and K08-3utr for *K08F9.1* and Y37-5utr and Y37-3utr for *Y37A1A.3*. The cDNA amplicons were inserted into the pCR-blunt II-TOPO vector (Invitrogen) to form pCR-fgt1, pCR-R09, pCR-C35, pCR-F53, pCR-F48, pCR-F13, pCR-Y61, pCR-K08, and pCR-Y37 plasmids, respectively.

**Table 1:** List of Primers.

Primer Name	Sequences (5'-3')
fgt1-5utr	AATGGGTGTCAACGACCATG
fgt1-3utr	AACCTATACGTTTCGCAGTG
R09-5utr	CAAAGGCTAGACATTCTATAC
R09-3utr	TGATTCTAATCCCGACTATG
fgt1up-5sph	CATGCATGCTGCGATTTGGAGCGAATCAG
fgt1up-3	TTCTGCAAAAAAAATTGATTTTTTAGGAG
fgt1cdna-5	ATGGGTGTCAACGACCATGATG
fgt1cdna-3xba	GCTCTAGACTTCCTCTTCTCGAATTCGGCTTG
R09up-5sph	CATGCATGCTTCCGGACTAGCTAGCATAC
R09up-3	CTATCATTTTTATTTTGACTGGCAAC
R09cdna-3sma	GGGTGAACATATCCGTTTTCTCATG
fgt1g-5bcl	CGCTGATCAAATGGGTGTCAACGACCATG
ppd-3bcl	CGCTGATCACAGGGAGAAAGAGCATGTAG
R09g-5bgl	CGCAGATCTCAAAGGCTAGACATTCTATAC
ppd-3bgl	CGCAGATCTCAGGGAGAAAGAGCATGTAG
fgt1ds-5	GAAAGCGCGCTGAAAAAGCTC
fgt1ds-3	TATCTCCCATTTTTGGCTTCTCCTG
M13 forward	GTAAAACGACGGCCAGT
M13 reverse	GGAAACAGCTATGACCATG
C35-5utr	TAGGTTCGTCTATCACACC
C35-3utr	ACCCGCAAATAATTTGAATAG
F53-5utr	CAATACCTGAACCTATTTATTC
F53-3utr	AGAAGCATTAACCAACAACAG
F48-5utr	AGTTTCAGAACCAGTGACAAC
F48-3utr	TTATGCTGGCTGTCATTA
F13-5utr	TTTCGACTGGTGCATGTGGTG
F13-3utr	AGCGATTCAAAACAAAGTTGG
Y61-5utr	TTGCCTCAATAGACAGCCA
Y61-3utr	TGTCATACCCGCTTAAAATCG
K08-5utr	TGTACCTCCAGTAACTTTG
K08-3utr	TTAAATAGGTTTGATTGAATTC
Y37-5utr	TTACAGGCAACTGAATGTC
Y37-3utr	ACAAAAACGGGACGAGCC

*C. elegans* expression plasmids of *fgt-1* and *R09B5.11* were constructed in *C. elegans* expression vectors pPD95.77 and pPD95.79 (Addgene plasmids 1495 and 1496), which harbor the eGFP gene for expressing the FGT-1::eGFP or R09B5.11::eGFP fusion proteins. A 2 kb promoter sequence upstream of the transcription initiation site of each gene was also cloned into the vectors to direct the expression of fusion gene. The *fgt-1* promoter was amplified from *C. elegans* genomic DNA with the primer set fgt1up-5sph and fgt1up-3, and the *fgt-1a* full-length cDNA was amplified from the pCR-fgt1 plasmid with the primer set fgt1cdna-5 and fgt1cdna-3xba. Both amplicons were digested with *Sph*I and *Xba*I and ligated simultaneously into *Sph*I- and *Xba*I-digested pPD95.77 to form the *C. elegans* expression plasmid of *fgt-1* (pPD-fgt1g). The *R09B5.11* promoter sequence was amplified from the *C. elegans* genomic DNA with the primer set R09up-5sph and R09up-3, and the full-length cDNA sequence was amplified from the pCR-R09 plasmid with the primer set R09-5utr and R09cdna-3sma. These amplicons were used as the templates for overlap extension PCR amplification of the DNA fragment containing both the promoter and cDNA by the primer set R09up-5sph and R09cdna-3sma. This amplicon and the pPD95.79 vector were digested with *Sph*I and *Sma*I and were then ligated to form the *C. elegans* expression plasmid of *R09B5.11* (pPD-R09g).

To construct the *Xenopus* oocyte expression plasmids of individual genes, the full-length cDNA of each gene was respectively inserted into the pSP64T vector, a plasmid that contains 5’- and 3’-flanking sequences of the *Xenopus* β-globin gene [25]. The *fgt-1* cDNA was excised from the pCR-fgt1 plasmid using *Kpn*I and *Xho*I. The *R09B5.11*, *F48E3.2*, and *Y37A1A.3* cDNAs were excised from the pCR-R09, pCR-F48, and pCR-Y37 plasmids, respectively, using *Eco*RI. The *C35A11.4, F13B12.2* and *Y61A9LA.1* cDNAs were excised from the pCR-C35, pCR-F13 and pCR-Y61 plasmids, respectively, using *Sac*I and *Pst*I. The *F53H8.3* and *K08F9.1* cDNAs were excised from the pCR-F53 and pCR-K08 plasmids, respectively, using *Kpn*I and *Pst*I. The pSP64T vector was digested with *Bgl*II. The digested vector and individual cDNAs were blunted by T4 DNA polymerase (New England Biolabs) and were then individually blunt-ligated to form the expression plasmids of *fgt-1* (pSP-fgt1), *R09B5.11* (pSP-R09), *C35A11.4* (pSP-C35), *F53H8.3* (pSP-F53), *F48E3.2* (pSP-F48), *F13B12.2* (pSP-F13), *Y61A9LA.1* (pSP-Y61), *K08F9.1* (pSP-K08) and *Y37A1A.3* (pSP-Y37), respectively. The human GLUT1 plasmid in pSP64T, pSP64T-hGLUT1, was a generous gift from Gwyn Gould [25].

To observe *fgt-1* and *R09B5.11* subcellular localizations in *Xenopus* oocytes, *the fgt-1::egfp* and *R09B5.11::egfp* fragments were also inserted into the pSP64T vector. These fragments were cloned from *fgt-1::egfp* or *R09B5.11::egfp* transgene-expressed worms by PCR with the primer set fgt1g-5bcl and ppd-3bcl or R09g-5bgl and ppd-3bgl, respectively. The amplified *fgt-1::egfp* fragment was digested with *Bcl*I, and the *R09B5.11::egfp* fragment was digested with *Bgl*II. These fragments were then ligated into the *Bgl*II-digested pSP64T vector to form pSP-fgt1g and pSP-R09g.

### Generation of transgenic worms

Transgenic worms were generated as described previously [26]. The *rol-6d* pRF4 (generous gift from M. Koelle, Yale University) was used as a co-injection marker [27,28]. The pPD-fgt1g plasmid was microinjected into the wild-type animals with pRF4 at 100 µg/ml. The animals were then fixed with 10 mM sodium azide. Transgenic animals bearing the GFP reporter were selected by the roller phenotype and were observed under a confocal laser microscope (Zeiss LSM 510 META Laser Scanning Microscope).

### *Xenopus* oocyte harvest and injection of cRNA into oocytes

cRNAs of *fgt-1*, *R09B5.11* and hGLUT1 were synthesized by *in vitro* transcription from pSP-fgt1, pSP-R09 and pSP64T-hGLUT1, respectively, using the mMessage mMachine kit (Ambion). Oocytes were harvested from *Xenopus laevis*. The synthesized cRNA (2 µg/µL) or sterile water was injected into isolated mature oocytes (stage V and VI) following previously established procedures [17].

The use of *Xenopus laevis* in this study was carried out in strict accordance with the recommendations in the Guide for the Care and Use of Laboratory Animals of the National Institutes of Health. The protocol was approved by the Institutional Animal Care and Use Committee of the University of Vermont (#13-025).

### Glucose uptake, kinetic and inhibition assays

2-DG uptake and the kinetic and inhibition analyses of FGT-1 and R09B5.11 were carried out in *Xenopus* oocytes as described previously [17]. cRNA- or water-injected oocytes were incubated in Barth’s media [88 mM NaCl, 1 mM KCl, 2.4 mM NaHCO_3_, 0.82 mM MgSO_4_, 0.41 mM CaCl_2_, 0.33 mM Ca(NO_3_)_2_, 5 mM HEPES, pH 7.6] with 10 µg/mL penicillin and 10 IU/mL streptomycin (Gibco) for three days and then subjected to the uptake assay using 10 mM 2-DG containing 1 µCi ^3^H-2-DG. For the phloretin inhibition assay, 0, 10, 25, 50, 75, 100 or 200 µM of phloretin was added to the assays. For the substrate specificity assay, 30 mM of each inhibitor sugar was added to the assays, with L-glucose as a control. For the kinetic assays, increasing concentrations from 0 to 25 mM of 2-DG containing 3 µCi ^3^H-2-DG were used. For all experiments, ten or more oocytes were used in each assay, and the oocytes were incubated in uptake solutions for 15 min. Each experiment was carried out individually at least three times.

### Localization analysis of FGT-1 and R09B5.11 in oocytes

cRNA of the *fgt-1::eGFP* or *R09B5.11:eGFP* fusion construct that were transcribed *in vitro* from the plasmid pSP-fgt1g or pSP-R09g plasmid were injected into *Xenopus* oocytes. The plasma membrane localization of the GFP fusion proteins was observed three days after the injection under a confocal laser microscope (Zeiss LSM 510 META Laser Scanning Microscope).

### RNAi in *C. elegans*

A 112 bp DNA fragment of *fgt-1* exon 6 was amplified from the pCR-fgt1 plasmid with the primer set fgt1-ds5 and fgt1ds-3 (Table 1). The amplicon was inserted into the pCR-blunt II-TOPO vector to form pCR-fgt1ds. To make the dsRNA for RNAi, the DNA fragment was amplified from the pCRfgt-1ds plasmid with the M13 forward and reverse primers (Table 1), and the PCR product was then used as a template for transcribing the single-stranded RNA (ssRNA) using SP6 RNA polymerase (Fermentas) and T7 RNA polymerase (Promega). The double-stranded RNA (dsRNA) was formed with the complementary ssRNAs and treated with Turbo DNase (Ambion).

RNAi experiments were carried out by injecting the *fgt-1* dsRNA into N2 *C. elegans* as described previously [29]. The synthesized *fgt-1* dsRNA was injected into young adult worms. The progeny from embryos laid 12 to 24 hours after the injection were isolated and observed for the various phenotypes described below.

### Western blotting and immunostaining

RNAi efficiency was assessed by the semi-quantitative measurement of FGT-1::eGFP protein levels in *fgt-1::egfp* worms with *fgt-1* RNAi. Protein levels were analyzed using Western blot procedures as described previously [17]. Ten worms were collected in sample buffer [62.5 mM Tris (pH 6.8), 2% (w/v) SDS, 10% (v/v) glycerol, and 5% (v/v) 2-β-mercaptoethanol]. The samples were incubated in boiling water for 5 min, centrifuged, and the supernatant was applied for SDS-PAGE. The proteins were transferred to a nitrocellulose membrane and were incubated with an anti-GFP (B-2, 1:250, Santa Cruz biotechnologies) or an anti-actin (C4, 1:500, MP Biomedicals) primary antibody for 2 hours at room temperature and then with a horse-radish peroxidase-conjugated anti-mouse IgG secondary antibody (1:5000, Amersham) for 1 hour at room temperature. The membrane was finally washed and detected using the West-Pico chemiluminescent kit (Pierce).

For the whole-mount immunostaining, mixed-staged worms were settled on Poly-Prep slides (Sigma), fixed and permeabilized using freeze-cracking followed by methanol treatment [30]. An anti-IFB-2 primary antibody (MH33, 1:20, DSHB) was applied overnight at 4 ^°^C. The Alexa Fluor 555-conjugated anti-mouse IgG secondary antibody (1:250, Invitrogen) was then applied for 2 hours at room temperature. Immunostained animals were observed under a confocal laser microscope (Zeiss LSM 510 META Laser Scanning Microscope).

### Sudan black B staining

A saturated solution of the fat staining dye Sudan black B in 60% isopropanol was diluted to 50% saturated in 60% isopropanol. Wild-type or RNAi-treated *C. elegans* were collected and fixed for one hour in PBS and an equal volume of 2x MRWB buffer [160 mM KCl, 40 mM NaCl, 20 mM EDTA, 10 mM spermidine, 30 mM HEPES, pH 7.4, 50% methanol] containing 1% paraformaldehyde. After fixation, the samples were washed with PBS, and Sudan black B staining solution was added and incubated for 16 hours at room temperature. Worms were then washed with PBS containing 0.05% Tween-20 and were observed with an optical microscope. Images taken by the microscope were analyzed with ImageJ software 1.44p (http://rsbweb.nih.gov/ij/) to quantify the levels of Sudan black B staining. The experiment was repeated at least three times.

### Brood size and dauer formation rate measurement

For the brood size assay, ten F1 progenies of dsRNA- or buffer-injected worms were individually placed on NGM agar plates and were transferred to new plates daily. The number of hatched progeny was counted daily. The counting was stopped when the worm produced less than ten progeny per 24 hours.

The dauer formation assay was performed as described previously [31]. Five dsRNA- or buffer-injected worms were placed on individual NGM agar plates for 24 hours at 20 ^°^C for laying eggs. After removing the parent worms, F1 progenies were incubated for 60-72 hours at 20 ^°^C or 25 ^°^C. Dauer worms were scored visually, and scoring was confirmed using SDS. Worms were considered dauers if they survived a several minute incubation in 1% SDS.

Each experiment was repeated individually at least three times.

### Statistical analysis

For the uptake and kinetic analyses, any uptake in *fgt-1* cRNA-injected oocytes that was lower than three times the mean value of that of water-injected oocytes was considered to be injection failure and was disregarded from the assay. 2-DG uptake in *fgt-1* cRNA-injected oocytes was corrected by subtraction of the mean 2-DG uptake of water-injected oocytes at the corresponding concentration and incubation time. Statistical significance was determined by Student’s *t*-test, Tukey-Kramer honestly significant difference (HSD) test, and Dunnett’s one-way ANOVA, as indicated in the individual figure legends, and the analyses were carried out using JMP 8.0 software (SAS Institute Inc.). Plots and curve fitting analysis for the Michaelis-Menten equation were carried out using GraphPad Prism 5.04 (GraphPad Software Inc.).

## Supporting Information

Figure S1

Amino acid sequence alignments of human GLUT1-4 and *C. elegans* GLUT candidates. Alignments of the deduced amino acid sequences of *C. elegans* genes *encoding* FGT-1, R09B5.11, C35A11.4, F53H8.3, F48E3.2, F13B12.2, Y61A9LA.1, K08F9.1, Y37A1A.3 and human GLUT1-4 proteins were performed with the Clustal W program with open gap penalty = 10 and gap extension penalty = 0.05. Residues that are highlighted by a black shaded background represent absolutely conserved amino acids and the gray shading indicates eight or more conserved residues at those positions. Regions of presumed transmembrane domains (TM) are indicated by numbered dashed lines, and the functionally important residues for glucose uptake activity are indicated by the letter “F” at the top of the sequence alignments. In addition, the highly conserved amino acids are shown on the bottom of the sequence alignment.
(TIF)

Figure S2 Phylogenetic analysis of human GLUT1-4 and *C. elegans* GLUT candidates. Human class I GLUTs and *C. elegans* GLUT candidates were aligned with Clustal W and a phylogenetic tree was drawn. The scale bar indicates the relative branch lengths obtained from the Clustal W alignment result.
(TIF)

Table S1 Scores of BLASTP searches of human GLUTs against Wormbase. (XLSX)
