# Supplementary figures and images for: FGT-1 Is a Mammalian GLUT2-Like Facilitative Glucose Transporter in Caenorhabditis elegans Whose Malfunction Induces Fat Accumulation in Intestinal Cells
Source: PLoS One. 2013 Jun 24;8(6):e68475. doi: 10.1371/journal.pone.0068475 (PMC3691140; doi:10.1371/journal.pone.0068475)

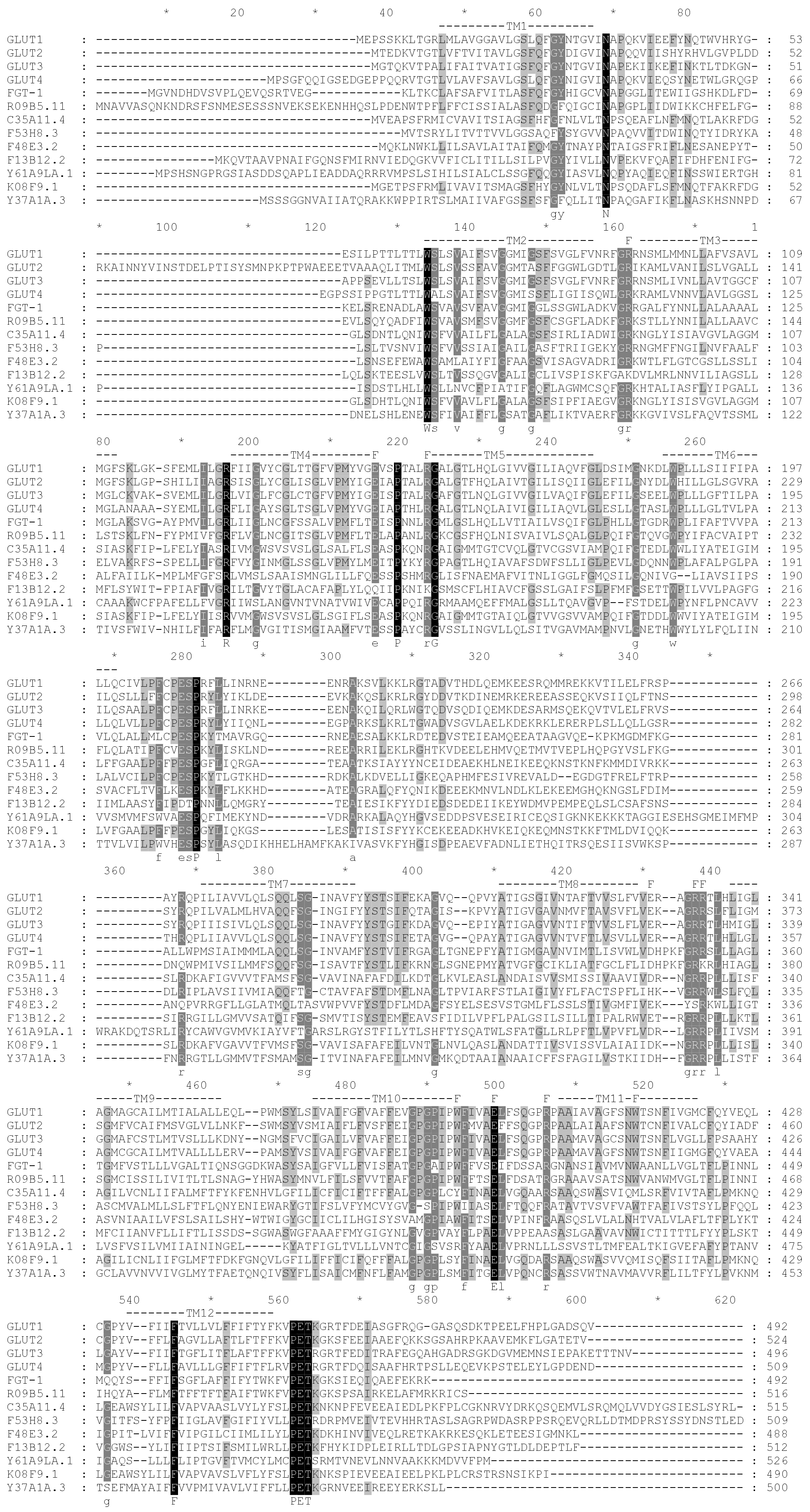

Supplement: Figure S1 — Alignments of the deduced amino acid sequences of C. elegans genes encoding FGT-1, R09B5.11, C35A11.4, F53H8.3, F48E3.2, F13B12.2, Y61A9LA.1, K08F9.1, Y37A1A.3 and human GLUT1-4 proteins were performed with the Clustal W program with open gap penalty = 10 and gap extension penalty = 0.05. Residues that are highlighted by a black shaded background represent absolutely conserved amino acids and the gray shading indicates eight or more conserved residues at those positions. Regions of presumed transmembrane domains (TM) are indicated by numbered dashed lines, and the functionally important residues for glucose uptake activity are indicated by the letter “F” at the top of the sequence alignments. In addition, the highly conserved amino acids are shown on the bottom of the sequence alignment. (TIF) [file pone.0068475.s001.tif]

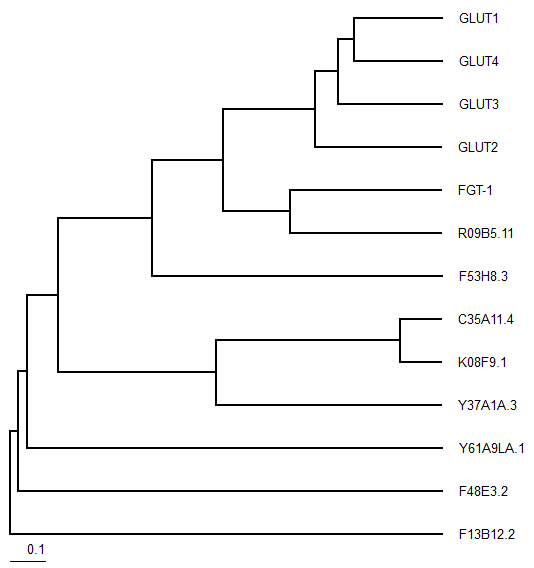

Supplement: Figure S2 — Human class I GLUTs and C. elegans GLUT candidates were aligned with Clustal W and a phylogenetic tree was drawn. The scale bar indicates the relative branch lengths obtained from the Clustal W alignment result. (TIF) [file pone.0068475.s002.tif]
